# ‘If I don’t Do It, I’m Out of Rhythm and I Can’t Focus As Well’: Positive and Negative Adult Interpretations of Therapies Aimed at ‘Fixing’ Their Restricted and Repetitive Behaviours in Childhood

**DOI:** 10.1007/s10803-022-05644-6

**Published:** 2022-07-04

**Authors:** Lynne McCormack, Sze Wing Wong, Linda E. Campbell

**Affiliations:** 1 University of Newcastle, Callaghan, NSW Australia; 2 School of Psychology, College of Engineering, Science and Environment, University of Newcastle, 2308 Callaghan, NSW Australia

**Keywords:** Restricted and repetitive behaviours, Autism spectrum disorder, Interpretative phenomenological analysis, Neurological diversity, Psychological growth

## Abstract

Restricted and repetitive behaviours (RRBs) are observed in many children presenting with characteristics of autism and are frequently the targets of psychological interventions. This study used Interpretative Phenomenological Analysis (IPA) to identify positive and negative interpretations from four young adults who received behavioural interventions in their childhood designed to ‘fix’ RRBs. Two superordinate themes were identified: (1) *Doubt, stigma and being fixed according to others*, and (2) *Embracing Authenticity*. They highlighted juxtaposed positions from exclusion, rejection, criticism, and self-doubt in childhood, to rejecting societal censure and embracing authentic growth in adult life. As adults, though the participants recognised themselves as neurologically different from others, they redefined themselves through a lens of neurodiversity, and therefore as not needing to be fixed.

## ‘If I don’t do it, I’m out of Rhythm and I can’t focus as Well’: positive and negative adult interpretations of therapies aimed at ‘fixing’ their restricted and repetitive behaviours in childhood

Restricted and repetitive behaviours (RRBs) are a central presentation in individuals diagnosed with autism spectrum disorder (ASD; henceforth referred to as autism). RRBs often cause significant challenges and barriers to individual adaptive learning, psychosocial and family functioning, and often attract stigmatising attitudes and behaviour from others (Barrett et al., 2004). RRBs are also linked to positive personal traits such as attention to detail; sustained focus; memory; and expertise. In addition, they are a coping mechanism for many in a complex, and sometimes confusing and stressful environment (Prior & Ozonoff, 2007). For instance, sophisticated interests involving particular expertise may benefit individuals by leading to niche employment opportunities in which individuals can utilise their special interests and expertise (Howlin, 2003). Therefore, this study seeks both positive and negative interpretations of adults previously diagnosed with autism and ‘treated’ in childhood for restricted and repetitive behaviours. As a qualitative study it explores the ‘lived’ experience from the insider’s perspective. Please note that we use both identity-first language (autistic person) and person-first language (person with autism) in this paper to reflect variability in the terminology preferences of the study participants and autism community.

Restricted and repetitive behaviours and interests as described by the Diagnostic and Statistical Manual of Mental (5th ed.; DSM-V; American Psychiatric Association 2013) are subdivided into four subtypes: (1) inflexible adherence to routines; (2) preoccupation with highly restricted and fixated interests; (3) stereotyped or repetitive motor movements; and (4) unusual interest in sensory aspects of the environment. MacDonald et al., (2007) suggested that self-stimulatory behaviours (SSBs) or ‘stimming’ are a specific group of RRBs which are readily noticed by others (e.g., spinning, rocking, phrasal repetition, or repetitive unrecognisable vocalisations). Some SSBs can be acutely harmful to the person (e.g., pinching or hitting self). Other stimming behaviours may be less noticeable and less socially problematic (e.g., rubbing, smelling, or sucking), and may provide benefits for emotional and cognitive self-regulation (Davidson, 2010; Kapp et al., 2019; Leekam, Prior, & Uljarevic, 2011). Again, these may also have the potential to cause harm when unrecognised as with lead poisoning through ingestion of paint fragments or sucking on rusty water piping. Regardless, stimming - often interpreted as problematic - has become a therapeutic target.

Generally, there is scant knowledge of the personal experiences of being the recipient of interventions for stimming behaviours. Joyce et al., (2017) provided the first evidence of young people diagnosed with autism and shared their perspectives on restricted and repetitive behaviours. The participants of their study considered restricted and repetitive behaviours as necessities in their lives highlighting that routines associated with these behaviours helped to reduce uncertainty associated with worry and anxiety. Similarly, autistic adults reflected on the impact of interventions received in childhood aimed at altering or removing stimming behaviours (Kapp et al., 2019). They described stimming as a self-regulatory mechanism that provided a calming rhythm enabling them to cope with sensory overload, an internal flood of thoughts, or uncontainable emotions (Kapp et al., 2019).

Evidence suggests that people diagnosed with autism are neurologically hyper-responsive to sensory stimuli. For example, sensory responsive behaviours (i.e., extreme negative response to, or avoidance of, sensory stimuli) may be associated with heightened responsivity in the primary sensory areas and those areas related to emotion regulation and processing (i.e. orbital-frontal cortex, hippocampus and amygdala) (Green et al., 2013). Indeed, recent studies have found that sensory hypersensitivity is strongly related more broadly to RRBs, not only among autistic but also neurotypical children (Schulz & Stevenson, 2019; Fetta et al., 2021). As such, stimming, resistance to change, and a predilection for routine, may each serve as adaptive coping strategies in response to an overwhelming, novel, and ever-changing environment (Lawson et al., 2014; Pellicano & Burr, 2012). However, it is important to note that different types of hyper- or indeed hypo-sensitivities may be linked to different types of RRBs and serve different functions (Schulz & Stevenson, 2019; Fetta et al., 2021).

Increasingly, anxiety and arousal are recognised as having an impact on: (1) repetitive motor; and, (2) insistence on sameness behaviours; in autism (Joyce et al., 2017; Lidstone et al., 2014) including e.g., restricted routines and interests (Gotham et al., 2013; Rodgers et al., 2012). This suggests that although engagement in RRBs is associated with anxiety, and therefore can be interpreted as a symptom of anxiety, they may serve also as a coping response to attenuate anxiety and excessive arousal. Different types of RRBs may also serve different functions with Lidstone et al., (2014) reporting that parent-rated anxiety symptoms in children were significantly associated with children’s insistence on sameness behaviours, but not with the repetitive sensory-motor behaviours. Moreover, they found that the relationship between insistence on sameness behaviours and anxiety was mediated by sensory avoiding.

Despite RRBs serving as coping mechanisms, suggesting that non-injurious forms of stimming are adaptive (Kapp et al., 2019; Orsini & Smith, 2010), RRBs are often targeted in skill-based behavioural interventions (Patterson et al., 2010), as well as in classroom management (Pas et al., 2016). Often, this is the result of parents or teachers seeking professional help and interventions to reduce a child’s stimming and stereotypic behaviours (Kodak & Bergmann, 2020). This is due to unusual or ritualistic behaviours being viewed as problematic, and potentially stressful and embarrassing for parents and carers, especially if the stimming behaviours are noticed by others in public (Swaab et al., 2017). Indeed, the interpretation of such behaviours often contributes to stigmatisation, rejection, and exclusion in society of individuals diagnosed with autism, and their families (Kinnear et al., 2016).

Applied Behaviour Analysis (ABA) is the most ubiquitous early childhood intervention recommended by clinicians and therapists for children diagnosed with autism. It is considered to be the golden standard for the treatment of autism (Vismara & Rogers, 2010). ABA is a teaching method based on theories of learning and operant conditioning (Lovaas, 1987). It relies extensively on external reinforcement to increase a behaviour, both positive (e.g., edible rewards, verbal praise, or tokens) and negative (e.g., removal of aversive stimuli), and external punishment to diminish a behaviour, both positive (e.g., restitutional over-correction, negative practice, or physical restraint), and negative (e.g., time out procedure or losing a token). Therapists or clinicians who adopt a behavioural model or ABA principle not only intend to enhance language use, communication skills, attention, and social skills in children diagnosed with ASD, but also to modify or reduce self-injurious behaviours and behaviours that have been deemed as socially unacceptable (Kodak & Bergmann, 2020). Arguably, the ethics of conducting interventions for stimming behaviours remain controversial, questioning whether the perception of the observer, not the individual with the behaviour, is the issue.

Sandoval-Norton et al., (2019) criticised behavioural interventions that involved long-term treatment to condition a child to stop stimming or obey commands such as ‘quiet hands’. Such treatment protocols evolved without an apparent understanding of the function of RRBs for autistic individuals with their neurological uniqueness. The question remains as to whether the use of change interventions for RRBs is irresponsible and possibly abusive. Interventions that involve various punishments and rewards without providing a meaning of the task that the individual is being asked to do, or not to do, can have negative impacts on individuals’ intrinsic motivation, self-esteem and self-efficacy to engage in the task (Sandoval-Norton et al., 2019).

Furthermore, there is evidence of increased post-traumatic stress in autistic individuals who have been exposed to ABA therapy (Kupferstein, 2018). Nearly half of the autistic adult and autistic child participants in Kupferstein’s study who were exposed to ABA early childhood intervention, noted posttraumatic stress symptoms. These included intrusive symptoms (e.g., nightmares of the traumatic event and flashbacks), persistent avoidance of trauma-related stimuli, negative alterations in cognition and mood associated with the traumatic event, and marked alterations in arousal and reactivity associated with the traumatic event (Kupferstein, 2018). More recently, qualitative evidence supports these findings further raising concerns regarding the long-term negative impacts of ABA therapy on the wellbeing of autistic adults (McGill & Robinson, 2021). Despite the evidence described above, the principle of ABA or behavioural model remains widely touted as an effective intervention for individuals on the autism spectrum, and the tenets of ABA have been integrated into other change interventions (Sandoval-Norton et al., 2019).

Despite the potential for negative psychological impact from exposure to behavioural interventions for RRBs, over time, many individuals redefine those experiences as being the trigger for greater self-determination and positive change in their lives e.g. (1) enhanced relationships, improved altruism, and compassion toward others; (2) changed self-perception with a greater acceptance of personal limitations and vulnerabilities; and (3) changed life philosophy with an appreciation of each new day and realisation that life is finite (Tedeschi & Calhoun, 1996; Joseph & Linley, 2005; McCormack et al., 2017).

In action, posttraumatic growth does not merely define a return to pre-trauma functioning, but the use of cognitive processing to incorporate the trauma-related information into a framework of new meaning post event (Zoellner & Maercker, 2006). Drawing on the work of Joseph & Linley (2005), the process theory of growth out of adversity posits that we have an innate human motivation to re-evaluate experiences toward an improvement in well-being and sense of fulfillment.

Nevertheless, there remains scant information regarding positive or negative outcomes from RRBs interventions in childhood and the subsequent social stigmatisation of their use in those with Autism. Therefore, using Interpretative Phenomenological Analysis (Smith, 1996), this idiographic study explores positive and negative subjective interpretations of adults diagnosed with autism reflecting on their childhood. Particularly, it seeks to understand how they perceive their RRBs or stimming behaviours, and any strategies or interventions that were introduced by others to ‘fix’ those behaviours during childhood.

## Method

### Participants

Four adults, diagnosed with autism, participated in the study. One reported a comorbid diagnosis of mild intellectual disability during childhood, but no longer met classification for intellectual disability (DSM, 2013). Of these, two participants were diagnosed using the Autism Diagnosis Interview-Revised (Rutter et al., 2003; Lord et al., 1994), while two participants reported that they had received clinical assessment and diagnosis by a paediatrician or clinical psychologist (4th ed.; DSM-IV; APA, 1994; 4th ed., text rev.; DSM-IV-TR, 2000, APA 2000). The mean age of the participants was 25 years (*SD* = 3.16), with ages ranging between 22 and 29. Only one of the four participants was female. Two of the participants self-identified as Anglo Australian, one as Aboriginal Australian and one as Asian. All participants experienced interventions or strategies, addressing RRBS, from clinicians, teachers, or their parents during childhood. Three of the participants reported that their mothers or parents first noticed characteristics of autism, one reported that characteristics were first noticed by his kindergarten teacher. The participants’ age of parental or carer concerns ranged between three and seven years of age, and their age of diagnosis ranged from four to 11 years old. Two of the participants studied at mainstream school with some assistance, one studied at mainstream school with no special assistance, and one studied at a specialised unit within a mainstream school. Table 1 presents a summary of the participants’ demographic information and additional information regarding levels of education and received interventions.

**Table 1:** Participants Demographic Characteristics Table

	Loretta	Jonathan	Stephen	Tobias
Who first noticed the signs of ASD?	Mother	Mother	Kindergarten teacher	Parents
Age of parental or carer concern	7	3	6	3
Age of ASD diagnosis	7	4	6	11
School Type	Mainstream – with some assistance	Mainstream – no special assistance	Specialised unit within a mainstream school	Mainstream – with some assistance
Highest level of education	Other (not specified)	University Student	University Student	Bachelor’s Degree
Received Intervention	CBT, ACT, DBT, occupation therapy, and parental intervention	CBT, parental intervention, and school-based intervention	Parental intervention, unspecified intervention conducted by psychologists, ASPECT, and school-based intervention	Parental intervention, unspecified behavioural intervention, and school-based intervention

*Note.* CBT – Cognitive Behavioural Therapy; ACT – Acceptance and Commitment Therapy; DBT – Dialectical Behavioural therapy

Self-identification and clinical assessment excluded potential participants from the study with current intellectual disabilities, sight or hearing impairment and acute mental distress (e.g., psychosis) to avoid conflating past and present treatment protocols that included multiple treatment regimes. Regarding the terminology used in this study, three of the participants preferred identity-first terminology, such as ‘autistic person’ or ‘autistic individual’. One participant did not reveal their preference.

### Data Collection

Following university human ethical approval, participant recruitment occurred by placing flyers around a local university campus, online support groups for adults identifying as autistic (or diagnosed with autism), and word of mouth, a common recruitment strategy of IPA studies known as snowballing. Prior to each interview, the interviewer sent a demographics form, study information letter, pre-consent form, and semi-structured interview schedule to the participants that allowed participants ample time (5 days) to reflect on the phenomenon being explored. The interview schedule was developed following a funnelling technique of IPA, which was designed to move the interview from general to specific interpretations. Two of the interviews were conducted via Skype, and two face-to-face interviews were conducted at the university. The interviewer discussed potential risks and benefits of participation with each participant prior to interview. Local counselling service phone numbers were provided to participants for post interview psychological support if needed. The average interview length was 74.25 min (*SD* = 12.33). All the interviews were audio-recorded and transcribed verbatim by the interviewer. All data were de-identified.

### Analytic Strategy

The current study aimed to access rich and reflective personal accounts regarding the participants’ experience of RRBs and interventions addressing these during childhood. Unlike positivist-nomothetic research, interpretative phenomenological qualitative studies are not constrained by predetermined hypotheses (Smith et al., 2009). From a critical realism stance, IPA acknowledges that individuals tend to be influenced by prior beliefs and experiences as interpretations are subjective (Blaikie, 2000). Hence, each narrative account uniquely represents the individual from an homogenous group who has experienced the same, but poorly explored, phenomena.

Methodologically this qualitative study was underpinned by phenomenology, hermeneutics, and idiography (Smith et al., 2009). For example, IPA as a phenomenological approach focuses on the exploration of human lived experience, and sense-making of personal meaning that individuals bring to their lived experience in a particular context (Smith, 1996). As an idiographic approach IPA details an individual’s unique experience. The interview is conducted with high sensitivity plus an in-depth exploration of clarity concerning the individual’s interpretation (Smith et al., 2009). Thus, IPA researchers engage in a double hermeneutic reiteratively engaging with the participant as they strive to give meaning to their experiences (Smith et al., 2009).

### Data Analysis

Each interview was analysed independently adhering to the four-stage process suggested by Smith et al., (2009). First, two researchers read each script repeatedly and wrote down notes of initial thoughts. Second, each produced a comprehensive set of notes, which involved more detailed comments and specific themes with a clear phenomenological focus. Third, the researchers developed emergent themes through mapping the connection and patterns between the exploratory notes, clustering themes, and ensuring each theme represented the participant’s own words from the transcript to prevent experimenter bias. Finally, these researchers engaged in joint analysis robustly discussing all emergent themes all the transcripts finally agreeing on main themes identifying convergent and divergent connections.

### Credibility and trustworthiness

Credibility and trustworthiness in qualitative research correspond with the concepts of validity and reliability as measured in nomothetic research. Unlike nomothetic research, IPA seeks authenticity of data, with credibility and worthiness achieved via continuing audit trails. Data is analysed first by an independent audit, and second through joint robust discussion to establish the final themes supported by rich data extracts (McCormack & Joseph, 2018). Detailed audit trails consist of initial notes on research questions, a research proposal, an interview schedule, audio recordings, transcripts, tables of themes, thematic definitions, a draft report and a final report (Smith et al., 2009). The first two authors independently analysed the data and then engaged in a robust discussion regarding their independent interpretations until convergent and divergent themes were substantiated by rich data extracts. As such, independent auditing delivered one account of the data that demonstrated the presentation of evidence and internal coherence (Smith et al., 2009).

The quality and validity in qualitative research can also be demonstrated in a number of ways (Smith et al., 2009). Firstly, a researcher demonstrates sensitivity to context and a close awareness of the interview process by showing empathy, recognising interactional difficulty, putting participants at ease, and navigating the convoluted power play between the researcher and experiential experts. Secondly, commitment and rigour occur through a high degree of attentiveness to the participant during data collection, the appropriateness of the sample, the quality of the interview and thorough and systematic analysis. Thirdly, the researcher endeavours to enhance transparency and coherence through a clear description of the methodology, a logical and consistent argument, and an appropriate degree of fit between the previous research and underlying theoretical assumptions.

### Author’s perspective

During this IPA investigation, researchers actively and consciously acknowledged and scrutinised biases and presupposition to prevent overshadowing interpretations with authors’ biases. Nevertheless, they brought experience and knowledge to the interpretation (Smith et al., 2009). The first author is a clinician and researcher whose interests focus on the interface between complex trauma and posttraumatic growth. She is extensively published in IPA. The second author is a psychologist who has experience in counselling and behavioural therapy with individuals diagnosed with autism. The third author is a clinician and researcher who has significant experience in undertaking research with individuals with developmental disorders including autism. The first and second authors brought perspectives from their experiences to the process of data analysis, but also challenged and questioned each other’s partialities with neutrality and insight throughout the investigation.

#### Community involvement

There is no community involved in this study.

## Results

Two superordinate themes (1) *Doubt, stigma and being fixed according to others*, and (2) *Embracing authenticity*; overarch five subordinate themes. These two patterns of response emerging from the subjects reflect the complexity of self and other’s judgement of self, and a desire to stay true to themselves. *Doubt, stigma and being fixed according to others* encompasses: (a) what is normal? (b) not good enough; and (c) forced to assimilate. *Embracing authenticity* is inclusive of (a) the secure base and (b) growth out of diversity. It reflects the participants’ journey facing accusations of abnormality against their ‘unusual’ behaviours, including stimming and insistence on sameness behaviours. Although their RRBs make them distinctive from what is regarded as neurotypical individuals, participants do not perceive that they need to be ‘fixed’. These participants face pressures to conform within a society that is guided by a set of norms or expectations of behaviours. Exclusion, rejection, criticism, and self-doubt continue to threaten their self-esteem. Despite these challenges, participants attempt to make sense of their RRBs and other neurotypical individuals’ reaction from their own perspectives. *Embracing authenticity* reveals the positive impact of family support, social acceptance, self-acceptance, and therapeutic alliance. These aspects are quintessential to the participants’ personal growth and convey a sense of hope and thankfulness for exposure to experiences and significant people who have positively contributed to their lives.

## Doubt, stigma and being fixed according to others

### What is normal?

In this theme, we explore the participants’ engagement with therapists as children initiated mostly by teachers and parents whose perception is that the participant has a disability and needs intervention to fit in with others, “mainstreaming us to the society”. Four out of four participants do not perceive that they need “fixing” but do recognise that they need help to “operate in the world”. However, with others perceiving their unusual behaviours and interests as not normal, parents sought help:

My mum started noticing I wasn’t meeting the criteria for normal children … you’ve got disability, you need some treatment …^1^ which didn’t really bother me as a child. (Loretta)

As children, they accept adult decisions - “slowly accept that I have a disability” but over time as they move into adolescence and early adult life, the complexity of trying to function in a world defined by behaviours, foreign to the participants, anxiety and mental health stress become their priority for treatment:

They don’t really address my main issue, it’s wasting time, I feel they are totally ignoring me. (Tobias)

Eventually, these participants recognise that the world perceives them as ‘abnormal’ and not the same as those described as neurotypical. “The interventions, they are just reminding me … the embarrassment”, leading to feelings of not belonging, isolation and grief:

When I started to see a psychologist, I’m like, okay I am not like everybody else, and I started realising it … I started to be very removed from everybody else … very isolated from people. (Stephen)

In adult life, for Loretta there is a determination to seek professionals who empathize with her struggle brought about from trying to adapt to ‘neurotypical’ and the mental distress this caused:

You need people … who can actually understand that there’s something serious going on … if you get the right treatment … you stop the unhealthy behaviours (self-harm behaviours)^2^. (Loretta)

Three out of four participants speak of the ever-present visceral distress as well as the mental health distress, not from their stimming behaviours but from the imposition of others’ perception of their behaviours. One participant speaks of shortness of breath and feelings of suffocation in attending therapy designed to help him function well within society rather than thrive as a unique individual:

I feel very nauseated, like vomiting, literally within me … it’s just … upsetting … totally upsetting. (Tobias)

When there is a mismatch, there is doubt about the use of medical treatment:

At first, they put me on antidepressants, it’s helping with like my emotions swinging but it’s not actually like fixing the problem. (Jonathan)

Two of the participants speak about accepting therapeutic intervention until the original treatment no longer met their needs. Trusting ‘self’ interpretation, each participant begins to investigate the core problem:

Probably would have worsened my condition over the years, or my ability to deal with my condition. (Jonathan)

Instead of blindly following the original treatment plan, one participant takes a courageous step forward to work with his stimming behaviours:

“Once I figured that out, then I was like, okay we need to figure out how to actually deal with it. (Jonathan)”

The ‘self’ interpretation continues, and Jonathan recognises his stimming behaviours as a rhythm to maintain his train of thoughts:

I do it every now and then to just keep a rhythm … if I don’t do it, I’m out of rhythm and I can’t focus as well …When I stop doing it … it messes with my thoughts and messes with the rhythm. (Jonathan)

Another participant emphasises the importance of stimming behaviours and identifies such behaviours as a “coping mechanism for living”, and that some stimming behaviours do not necessarily require intervention:

When a kid is just tapping or flapping hands, I don’t really think that’s a problem and I don’t think it should be treated like a problem. (Stephen)

Traditionally, stress has been thought to be a trigger of stimming behaviours therefore intervention has focused on stress management intervention. One participant argues against this sentiment:

I don’t think the stress is the reason why I do it, I just do it. I think it’s the opposite, I do it, so that I don’t feel stressed, because if I don’t do it, then I get agitated. (Jonathan)

### Not good enough

Within this theme, the participants share their vulnerability and the eroding of individuality. Others see the participants’ behaviours as problematic throughout their childhood and in adult life. Eventually, others’ expectations cause the participants to emotionally spiral. They feel the negative weight of being ‘not good enough’:

I just felt that, well, maybe I’m not a good person … and I do not matter or that I am just lesser than their expectation. (Tobias)

‘Autistic individuals’^4^ struggle to live up to other people’s expectations. For Stephen, he held on to a belief that was passed down from his father - “autism is a condition, but it’s not an excuse not to try your best in life”. Adopting the perspective of his parent, Stephen set a high standard for himself and firmly declined any assistance at school. Tobias talks of the enormous effort he made during the treatment, which resulted in depression, confusion, exhaustion, and leaving him feeling drained:

After the treatment ... I just felt down and wanting to do nothing at all and lost. They took the energy away from me … they also took away the life of me. (Tobias)

Other people perceive the participants’ stimming as deficient and problematic and attempt to rectify their behaviours. Consequently, one participant conveys a feeling of guilt and develops an over-generalising conclusion:

The stimming … is a coping mechanism that they thought … needed to be corrected – whatever I did was wrong. (Tobias)

Other people’s perception of participants’ RRBs turns into a stigmatisation of RRBs, which has manifested in prejudice and discrimination:

I find it sad that instead of trying to understand the disability, people just jump straight to making fun of you, mocking you, saying things bad to you, spreading information saying that we need to be cured. (Loretta)

One participant comprehends the existence of prejudice and discrimination as something that likely stems from the neurotypical individuals’ anxiety:

I think people become very anxious about it; I mean people are being very defensive, very protective. (Stephen)

As the stigmatisation continues, one participant communicates the idea of being ostracised by the community and the magnitude of feelings he experiences:

… alienated, … I think they kind of have an impression that I got to be treated very differently all the time … I kind of feel painful - people treated me differently. (Stephen)

Some people see participant’s RRBs as a sign of immaturity, and this perspective has an ongoing impact on the participant’s self-esteem and self-image, persisting into adulthood:

I will still remain a man-child … the perception of other people’s - the perception of my behaviours (i.e. spinning, hands flapping and hoarding), was and still is, still something that makes me insignificant and immature. (Tobias)

### Forced to assimilate

This theme represents the difficulties and struggles experienced by these participants as they grow up in a society that embraces informal rules that govern behaviours in different settings and communities. Being on the autism spectrum, four participants speak of a sense of “living in a world that wasn’t made for us”. Memories of their time at school are evoked, where a sense of discomfort about the change of routine is recalled:

One of the key things of ASD is change doesn’t really make you feel good … having seven or eight different teachers every single year, it’s kind of just like overloaded … just like I am getting hounded by eight or nine teachers for having the same behaviours and not being able to focus. (Jonathan)

In addition to the intolerance of changes, participants face challenges from living in a world that criticises, rejects, and excludes their RRBs.

…between acting like myself and potential getting rejected… it was really difficult, like the whole tapping thing, I will get in trouble at school (Jonathan)

Three out of four participants speak of the struggles in exploring the environment and engaging conversation with other people. The sense of constraint, minimisation, and the yearning for freedom, is metamorphic:

If I don’t get to stim, and then I feel that I’m not able to go to the places that I really wanted to go, I feel restricted …when I feel restricted, or I feel very small and confined in a very restricted space, maybe I can flap my wings out of the small space that I have and go out to a bigger world … maybe I have something bigger that I can look forward to, to move around. (Tobias)

Metaphors are helpful in bringing understanding:

I don’t really wanna call it a disorder, it’s a neurological condition. It’s the way my brains physically structured. It just different. It’s like everyone else is on FM radio for their brain and I am still on the AM radio for my brain. (Stephen)

Rumination or repetitive thinking is a common cognitive processing difficulty associated with autism. One participant describes a delay in thought processing during a dynamic conversation:

I wanna have a thoughtful opinion, but then I start thinking … by the time I am ready, … they’re talking about the next thing, I am like, “argh”. (Jonathan)

As the participant recognises the delay in response time during a reciprocal conversation, he skilfully adapts to the dynamic conversation using some pre-programmed response:

When I am able to react quickly it’s because I’ve already like pre-saved that response in my head. (Jonathan)

Meanwhile, engaging in a conversation with unfamiliar people can be challenging for one participant:

I was made to do a group assignment, which was the worst, cause I have to communicate with people that I don’t know and it makes me anxious, but once I get to know them, I get quicker responding. (Jonathan)

Despite the feeling of being isolated and forced to assimilate, this participant reflects on his communication skills and thinks about ways to improve his skills. A sense of hope motivated the participant to remain committed to practicing his communication skills:

I could’ve said something meaningful and constructive, and we’d have been on the same wavelength, but I didn’t know, cause I haven’t spent that much time with you (the person he interacted with), but I’ll know next time. (Jonathan)

Participants learn to adapt to this ‘process of assimilation’; as they perceived the world they are in honours assimilation rather than the inclusion of difference. Battling others’ perceptions and attempts to integrate neurotypical behaviours, participants cleverly learn to disguise themselves as neurotypical:

They don’t allow us to be ourselves, so sometimes I have to put on a bit of mask when you are out in the world. (Stephen)

One participant transformed his routine and ritualistic behaviour into a means of connecting with other people. He speaks about feeling annoyed when someone else sits on the seat that he usually sits in, and that he feels uncomfortable when he is not in that seat. This preference has facilitated some meaningful interactions with his friends in class who learn about his preference. Instead of being rejected, by educating his friends, he found that he could engage in humour even if frustrated, which he came to recognise as friendship:

If someone else sit on my seat, that annoys me, cause I am like, “argh, that’s my seat.” they would move the seat, … “Still the same seat”, then I was like, “But it is not on the same spot, you don’t understand” … They’re just messing with me. (Jonathan)

## Embracing authenticity

### The secure base

This theme illustrates the support participants have received from their friends, families, and clinicians. Despite the challenges and difficulties in adapting to the social norms, two of the participants experience a sense of belonging and security within a group of people who embrace the differences:

I have a lot of friends who understand me and that’s all I really need, I don’t need a big group of friends, I just need people who’ll stand by me no matter what. (Loretta)

Not only does the quality of relationship include a genuine acceptance, but the relationship also involves appropriate boundaries and mutual respect:

it’s like the five or six close friends that I do have … the fact that they know who I am and then they know like what rattles me up and what doesn’t, but they know where the line is, which is great. (Jonathan)

The quality of relationship is also manifested in sharing thoughts with friend who share similar experiences, thus promotes the participant’s sense of self-worth:

My friend has a tendency to balance the thoughts, so she would like to consider both non-autistic person’s point of view and her own experience, which is quite similar to mine… after meeting my friend that I felt maybe there’s something worthwhile in me. (Tobias)

The quality of friendship outweighs the quantity of friends:

If I only have like five or six people, but they accept me for who I am, then I am perfectly fine. (Jonathan)

Two participants speak of the importance of family support, in which their families have provided a secure base from which to grow and explore the world:

You know, your family says you’re not horrible, you’re not a burden, you’re not stupid, you’ve got to realise how beautiful you are and talented you are. (Loretta)

Parents become advocates for their child with autism, notwithstanding that they may not acquire professional knowledge about autism:

Because my parents knew what ASD was about, but they’re not professionals … they couldn’t explain on a psychological level, why that was the case, they just like, “oh you feel better when you do this, I don’t know why, but we know that you like doing this, so we won’t try to make you do this”. (Jonathan)

A stable therapeutic relationship nurtures self-awareness and acceptance:

“Having discussions with the psychologist every week we slowly came to a conclusion that, ‘oh this isn’t working, but I think you’ll be better with this’, so constantly have that rapport … to help me figure out more what was making me feel bad in terms of emotion and stress and what made me feel good in terms of emotion and stress. (Jonathan)”

A positive experience from the therapeutic intervention allows one participant to become more receptive to assistance. There is a sense of active acceptance without dishonouring the self:

I realise that sometimes I need extra help, and that’s okay. I don’t need to push myself … trying to keep up with everybody else, because I am not like everybody else, and that’s not a bad thing. (Stephen)

### Growth out of diversity

A common theme within these stories is the process of participants consciously recognising the value of their RRBs and accepting ‘self’ as different from others. Most remarkably, they speak of freedom of choice following a sense of self-acceptance. In addition, the intervention becomes a window of self-insight associated with the perception of stimming behaviours:

It (the intervention) opened up my sight and my experiences, my senses to the things that are associated with the perception of stimming. (Tobias)

Despite the intervention having been a painful experience for Tobias, he transforms the pain into a strength that motivated him to pursue his dream:

I guess the painful and terrible experiences from school had strengthened me … they strengthened me, and I was still able to keep my dream, where I can experience liberation and a lot of positive support for what I do and how and what I am. (Tobias)

As participants grow and become gradually conscious of their RRBs, they try to make sense of their behaviours. Tobias illustrates that stimming behaviours give him a sense of freedom, anticipation, and healthy dissociation from aversive experiences:

… very anxious waiting for something to happen, and then I will just walk and walk around in circles while waiting ... when I spin around, I won’t feel like I am staying in a certain place, so it won’t feel like waiting ... it feels like moving all the way to where I wanted to go … I feel lighter, I feel not being restricted, it feels like I am lifting off to somewhere … it feels like I am fluttering around like a butterfly! (Tobias)

The stimming behaviours are seen as a time machine that allows Tobias to travel forward to his anticipation or backwards to pleasant moments:

It takes away the scariness and anger - at least I am doing something that I am looking forward to … It helps me temporarily forget the nasty stuff - to think of the beautiful moments without really considering what other people around me think. (Tobias)

Participants courageously acknowledge that the self and others behave differently:

In terms of the acceptance, I just realise that I do things that is quirky in nature compared to like my friends who are just perfectly fine, I just tend to do things that may seem a bit weird to the normal individual and society. (Jonathan)

This sense of self-acceptance has encouraged the participant to choose to engage in RRBs that have the least impact on others:

I only knew that I was different, I knew that I behave differently but I just tried to ignore it, I know it’s not ‘normal’, it’s not a burden though, so I don’t see the problem … I try not to create drama now. (Loretta)

This sense of self-acceptance also allows the participant to make a choice that helps him to reach his academic goal:

Because at high school I refused to accept teacher’s aids … but I started to accept the aids at the university. (Stephen)

One participant speaks of honouring his uniqueness. The purpose of life is being manifested in his uniqueness:

I become more conscious that what I do maybe has … a purpose… it is not because of my weakness or my nastiness … it could be because of my own uniqueness. (Tobias)

The power of self-acceptance has brought personal growth and intrinsic transformation:

Realising my ‘disability’ and the full potential of, not as much as using it as a weapon and like attacking people but using it as kind of like a gift and, like, telling other people like me that you know it’s beautiful to have a disability. (Loretta)

## Discussion

This study sheds light on the subjective interpretation of four participants diagnosed with autism who experienced interventions targeting their restricted and repetitive behaviours. Results indicated two superordinate themes 1) *Doubt, stigma and being fixed according to others*, which overarched three subordinate themes: (a) *What is normal?* (b) *Not good enough; and* (c) *Forced to assimilate*, and 2) *Embracing Authenticity*, which overarched two subordinate themes: (a) *The secure base; and* (b) *Growth out of diversity*. From the adult perspective, the participants reflected on their childhood characterised with confusion, distress, social isolation, and self-doubt. Once signs and symptoms of autism spectrum disorder, as defined by the DSM (American Psychiatric Association, 2013), were noticed by participant’s parents or teacher, participants received a range of interventions, special education and parenting strategies targeting their RRBs. These themes illustrated the enormous impact of the stigmatic social, medical, and educational systems and associated interventions on participants’ self-esteem and personal assumptions. These participants, however, did not identify themselves and their unusual harmless behaviours as a problem that required fixing. Importantly, results from this study suggest that for these participants, RRBs require understanding for their unique contribution to the participants’ functioning in the world. Together with the participants’ family and friends who genuinely accepted them, participants have redefined their sense of flawed self to one that is uniquely neurotypical promoting psychological growth. This allows them to reject boundaries established by the bias that neurotypical is normal and non-neurotypical is pathological and therefore disordered.

The participants in this study spoke of passively receiving the diagnostic label and associated interventions during their childhood. As children, participants recalled not recognising their functioning and behaviours as distinct from the other neurotypical individuals. Nevertheless, their RRBs were identified as ‘problem behaviours’ broadly defined as behaviours that are not socially acceptable, negatively impacting their functioning, and physically dangerous (Kodak & Bergmann, 2020). ‘Problem behaviours’ became one of the main reasons for referral to intervention and a source of parents, caregivers or teacher’s stress and concerns. Participants recalled the experiences of being removed from a standard classroom, seeing a professional for behavioural intervention, receiving medical treatment, and being involved in specialised autism education setting. Many described feelings of isolation, confusion, restriction, suffocation, and sadness during the interventions. Participants in this study recalled that others’ judgement and perception of their RRBs led participants to wonder *what is normal?* Questioning ‘normal’ guided by social norms and the medical model, participants challenged neurotypical bias suggesting a more holistic shift in thinking and an inclusive concept of neurodiversity. As such, participants suggested that a spread of acceptance of individual functioning along a continuum would preclude a need to be cured, and highlight diversity of behaviour, some of which might benefit from assistance, integration, and accommodation.

As the social stigma associated with ‘not normal’ behaviours continued, these participants recalled bullying experiences leading to negative self-evaluation and self-blame (Kinnear et al., 2016). In this context, participants experienced feelings of guilt, sadness, and the pain of being alienated. Previous research has found that youth diagnosed with autism are more likely to experience bullying compared to their neurotypically developing peers, especially for those who also reported being more socially anxious (van Schalkwyk et al., 2018). A participant of this study gave an insight into the interpersonal dynamic between autistic individuals who exhibit RRBs and the neurotypical individuals, suggesting that neurotypicals’ intolerance of diversity during interactions with those exhibiting RRBs may cause a rise in anxiety rather than the RRBs themselves. Nacoste (2010) examined individuals’ emotions and struggle in response to various social markers of difference, such as race, gender, ability, and religion suggesting that individuals often experience anxiety during the interaction with others who vary from them in terms of one or more of these social markers. Likewise, in addition to others’ perception of their RRBs and criticism against their RRBs, autistic individuals also shared experiences of intolerance of uncertainty living in a rapidly changing world and overstimulated world. These factors may induce a high level of stress, anxiety, and worry, which may be associated with the engagement of insistence on sameness behaviours (Lidstone et al., 2014; Wigham et al., 2015). Future research may consider the interaction between these two psychological processes.

One of the participants in this study categorised interventions for RRBs into two types: interventions for harmful RRBs (e.g. self-hitting or hand banging) and interventions for harmless RRBs. It is suggested that interventions for harmful RRBs could be effective as crisis management strategies to de-escalate potentially dangerous situations. On the other hand, others’ attempts at interrupting harmless stimming behaviours and differential reinforcement of alternative behaviour, were commonly experienced by the participants. Consistent with previous research, participants reported that these approaches effectively restrained and modified their repetitive behaviours deemed ‘problematic’, such as tapping objects, flapping hands, pacing in a circle and spinning (Patterson et al., 2010). However, the interruption interventions were implemented at the expense of participants’ internal processing. Individuals diagnosed with autism are often neurologically hypersensitive to sensory stimuli, they have trouble with filtering and processing sensory information and are often overwhelmed by stimuli in the environment that other people may not even notice (Green et al., 2013). As a result, autistic individuals may engage in sensory modulation behaviours to regulate their sensory experience (Green et al., 2013; Kapp et al., 2019). Similarly, the participants of this study recognised RRBs as innate coping mechanisms to overcome an overwhelming environment providing a calming rhythm to maintain focus. One participant highlighted that RRBs are emotional regulation strategies, and the restriction of RRBs causes agitation. Recent qualitative research supports the view that autistic adults see stimming (i.e., an RRB) as a self-regulatory behaviour with many positive effects experiencing negative consequences when they are forced to repress them (Charlton et al., 2021).

The subordinate theme *Forced to assimilate* reflected the boundaries and difficulties experienced by the participants in various social settings and during interpersonal interactions. They described the social norms and informal rules as restrictions against their RRBs. These participants came to realise that they are neurologically different from individuals considered to have had a neurotypical development. It has been proposed that people with autism skilfully developed strategies to disguise themselves as neurotypical in a stressful world (Dean et al., 2016; Tierney et al., 2016). Known as ‘passing as non-autistic’ (PAN: Libsack et al., 2021) this may bring advantages and deflect criticism. However, negative implications are recognised including pressure to conform to neurotypical social expectations, poor mental health e.g. internalised depression and anxiety, avoidance of supporting services, and significantly delayed, inaccurate, or lack of an ASD diagnosis that may bring benefits (Libsack et al. 2021; Miller et al., 2021; Cage & Troxell-Whitman, 2020). Although the difficulties in screening for ASD and a controversy over universal screening remain a challenge (McCarty & Frye, 2020), early diagnostic evaluation for ASD has become increasingly common which may potentially attenuate the issue of a delayed diagnosis (Pierce et al., 2019; Sicherman et al., 2021).

Despite the many challenges faced by the participates, this study provided evidence that growth out of adversity was possible for these participants. Through family support, genuine friendship and tailored interventions to address participants’ needs, each participant sought to overcome the criticism and aversive experiences. Consistent with work of Joseph & Linley (2005), participants demonstrated an actualising tendency towards psychological growth through a favourable and supportive psychosocial environment. They expressed gradual self-acceptance over time, resulting in a shift away from self-doubt and self-blame towards a new perspective of their RRBs. The sense of shame associated with the RRBs was reframed to free them from a restricted state of self-doubt. They recognised the potential of sensory enrichment and their uniqueness. Moreover, positive change in interpersonal relationships was identified. Participants spoke of opening themselves up to receive help and develop supportive friendships.

There are clinical implications from this study. These findings demonstrated that the four autistic participants who experienced distress associated with stigma, blame, rejection, and shame, interpreted trusting relationships as a springboard for self-valuing. As such, therapists are encouraged to assist autistic individuals to build self-awareness and acceptance and gain a better understanding of themselves in the world that engages with healthy self-esteem and self-image (Huang et al., 2017). Therapists can mentor trusting and supportive relationships within the therapeutic space and provide a safe place for their clients to explore an intervention suited to their psychological needs rather than being ‘fixed’, thus enabling them to confidently engage with self, others, and their life philosophy. As reported by participants, engaging in a collaborative therapeutic relationship facilitated growth, self-acceptance and awareness in this study. Spencer et al., (2019) suggested that a collaborative therapeutic relationship involved clients playing an active role in decision making, recognising the client’s expertise and building trust. Gifford & Knott (2016) suggested that diagnostic labels affect care staffs’ perception and attribution of challenging behaviours in individuals with development disorders or learning disability, and the diagnostic label also affects care staffs’ emotional responses. Therefore, therapists should be mindful of their unconscious bias and attribution towards neurological and developmental differences.

### Limitations

The current study has several limitations. As an interpretative study, researchers may have introduced bias associated with personal subjective experience during the process of data collection, at interview, or theme selection and analysis. Researchers have consciously sought to minimise such biases through robust discussions and rereading the original transcripts to ensure that interpretations and themes remained grounded in participants’ accounts. Considering autism is known as a ‘spectrum’ condition due to a wide variation in type and severity of presentation and individual experience, the participants in this study were predominantly identified as ‘high functioning’ although this is a controversial label, not adequately describing the social difficulties associated with autism. As the qualitative findings of this study are limited to the participants and cannot be generalised, they are limited also in shining a lens on the diversity of functioning in individuals with autism. Similarly, there were limitations due to the homogenous nature of the group of individuals with a diagnosis of ASD included in the current study. Further studies could seek intersectional experiences providing information on diversities of autistic identities and positioning e.g., gender, sexuality, ethnicity, race, social, and educational/intellectual status.

## Conclusions

In summary, our findings indicate that others’ perception of participants’ RRBs and certain restricted interventions induced confusion, frustration, distress, isolation, and self-doubts in these participants. Our participants expressed unpleasant experiences when their RRBs were being criticised and interrupted during therapeutic interventions. Importantly, results suggested that participants continue to bring meaning to their RRBs, their diagnosis, interventions, and the dynamics between themselves and other people. Moving away from receiving intervention passively, and growing from past experiences, these participants have come to actively be involved in understanding their RRBs, changing their perception of their RRBs and diagnosis, and developing meaningful relationships in their lives. Participants recognised the neurological difference between themselves and other people highlighting the concept of ‘neurodiversity’ with a non-pathological perspective of not needing to be ‘fixed’ (den Houting, 2019). Integration was thus a two-way shift of biases where differences were accepted and valued not pathologised. Crucially, this study highlights the potential for personal growth, even posttraumatic growth in those who have experienced traumatic distress through the well-meaning intervention of others, through acceptance. Clinical implications of this study include a) facilitating the development of healthy self-esteem and self-image irrespective of neurological diversity; b) developing a collaborative therapeutic relationship that values uniqueness; and c) being aware of unconscious bias and attribution of individual’ difference allowing for unconditional positive regard of self to flourish.

## Transcript Extract Notations

[…] indicates editorial elision where non-relevant material has been omitted.

^2^ ( text ) indicates explanatory text added by author.

^3^ [–] pause in speech.

^4^ ‘autistic person’ phrase identified as preferred by participants.
